# Harnessing natural killer cell effector function against cancer

**DOI:** 10.1093/immadv/ltad031

**Published:** 2023-12-21

**Authors:** Matthew D Blunt, Salim I Khakoo

**Affiliations:** School of Clinical and Experimental Sciences, Faculty of Medicine, University of Southampton, Southampton, UK; School of Clinical and Experimental Sciences, Faculty of Medicine, University of Southampton, Southampton, UK

**Keywords:** natural killer cells (NK cells), chimeric antigen receptor (CAR), immunotherapy, CAR-NK cells, cancer, NK-cell engagers

## Abstract

Natural killer (NK) cells are cytotoxic innate lymphoid cells that participate in anti-tumour and anti-viral immune responses. Their ability to rapidly destroy abnormal cells and to enhance the anti-cancer function of dendritic cells, CD8+ T cells, and macrophages makes them an attractive target for immunotherapeutic strategies. The development of approaches that augment NK-cell activation against cancer is currently under intense preclinical and clinical research and strategies include chimeric antigen receptor NK cells, NK-cell engagers, cytokines, and immune checkpoint inhibitors. In this review, we highlight recent advances in NK-cell therapeutic development and discuss their potential to add to our armamentarium against cancer.

## Natural killers of cancer

Natural killer (NK) cells are innate lymphocytes that develop in the bone marrow from common lymphoid progenitor cells. NK-cell maturity and function are critically dependent upon the transcription factors T-BET and EOMES [1] and following maturation they express a diverse repertoire of germline-encoded, non-rearranged surface receptors [2, 3]. This diversity in the expression of activating and inhibitory receptors allows for the recognition of infected or tumour cells through complementary mechanisms in a combinatorial manner. As such, NK cells are fine-tuned to recognize changes in the balance of signals derived from those receptors. There are several different inhibitory receptors including NKG2A, inhibitory killer cell immunoglobulin-like receptors (KIR), TIGIT, and LILRB1. NKG2A binds human leukocyte antigen (HLA)-E, and upregulation of HLA-E on cancer cells can inhibit both CD8+ T cell and NK-cell function via NKG2A [4]. In addition, the diverse family of inhibitory killer cell immunoglobulin-like receptors (KIR) predominantly engage with HLA-A, -B, and -C alleles. Thus, the loss of HLA class I ligands for inhibitory receptors such as NKG2A or the inhibitory KIR proteins on cancerous cells leads to loss-of-self inhibitory signals and NK-cell activation [2]. The interaction of KIR and NKG2A with their ligands on healthy cells is also necessary for the production of functionally competent NK cells (termed NK-cell education). These ‘educated’ NK cells possess a superior ability to kill target cells than those that do not express a receptor for self-HLA class I. Interactions of NK cells with HLA class I determine global NK-cell function via fine tuning the quantity of releasable granzyme B which is crucial for cytotoxic activity [5]. Furthermore, the non-classical HLA class I molecules, HLA-F, and -G can also modulate NK-cell function. Upregulation of HLA-G in cancer can inhibit NK cells through engagement of LILRB1 and is associated with worse outcomes, suggesting potential as a target for therapeutic blockade [6–8]. In addition, HLA-F, which binds the activating receptor KIR3DS1 as an open conformer, may also be upregulated in cancer [9] and this may paradoxically be associated with worse survival [10–12].

There are many activating receptors expressed by NK cells, and some examples of relevance to cancer immunotherapy include NKp46, NKp30, DNAM-1 (CD226), 2B4 (CD244), activating KIR, NKG2D, NKG2C, and NKp44 [2, 3, 13, 14]. In cancer, the ligands for NKG2D and NKp46 are stress-induced ligands such as MICA/B, ULBPs, and ecto-calreticulin [3, 15]. Both the activating receptor NKG2C and the inhibitory receptor NKG2A bind to HLA-E. HLA-E can therefore have a dual role in both the promotion and inhibition of NK cell function. The effector functions of NKG2C have largely been demonstrated in the context of viral infections, however, they may also operate in cancer as NKG2C+ NK cells have recently demonstrated potent cytotoxicity against HLA-C:KIR mismatched leukemic cells [16]. Through their expression of CD16, NK cells mediate strong activation in response to target cells bound with IgG1 antibodies and this provides NK cells with the ability to respond to tumour targeting antibodies via antibody-dependent cellular cytotoxicity (ADCC) [17, 18].

Upon target cell recognition, NK cells can rapidly induce target cell lysis through the secretion of granzymes, granulysin, and perforin into the immunological synapse. Importantly, NK cells are protected from autolysis by densely packed lipid membranes [19]. Due to their expression of FASL and TRAIL, NK cells can also induce apoptosis of target cells through the engagement of death receptors. In addition to these direct cytotoxic actions, NK cells have an important role in shaping the multi-pronged anti-cancer immune response via the secretion of chemokines and cytokines. For example, NK cells recruit conventional type 1 dendritic cell (cDC1) into tumours due to their secretion of the chemokines XCL1, XCL2, and CCL5. These chemokines can also promote DC differentiation and DC survival via FLT3 ligand, and can promote CD8+ T-cell recruitment and activation at tumor sites [20–23]. Furthermore, NK-cell secretion of IFNγ can upregulate MHC class I expression which is critical for neoantigen recognition by CD8+ T cells. Finally, NK cells can promote antibody-dependent cellular phagocytosis of tumour cells by macrophages [21, 22, 24, 25].

The important role of NK cells in the anti-tumour response is well established in a multitude of murine and *in vitro* models, and in humans was illustrated by a landmark study showing that superior NK-cell function are associated with reduced cancer incidence [26]. Accordingly, enhanced NK function and infiltration is associated with improved prognosis in multiple solid and haematological malignancies (reviewed in [21, 27]). NK cells are less frequent in tumours than T cells [24], and NK-cell function can also be rapidly (within 48-72 h) suppressed following infiltration into tumours [28]. Thus in some solid organ cancers, CD56dim CD16high HSP40+ tumour-asssociated NK cells display a dysfunctional state with reduced perforin and granzyme B [29]. Therapeutic strategies must therefore overcome NK-cell exhaustion and augment NK-cell persistence, infiltration, and tumour cell recognition to have durable anti-cancer activity (reviewed in [30, 31]).

Although members of the innate immune system, recent work has highlighted that NK cells possess features of trained immunological memory [32]. This is relevant for NK cells generated through viral infection, contact hypersensitivity, and cytokine stimulation with IL-12, IL-15, and IL-18 to generate cytokine-induced memory-like (CIML) NK cells. These CIML NK cells have been shown to possess improved persistence, expansion, metabolic function, and activation against tumour cells both *in vitro* and *in vivo* in multiple solid and haematological cancer models [33–38]. Based on these promising pre-clinical data, CIML NK cells are now under assessment in early-stage clinical trials for patients with solid or hematological cancers (NCT05470140, NCT02782546, NCT04634435, NCT04290546, NCT04024761). Furthermore, genetic engineering strategies have shown promise in the recapitulation of the features of CMV-adapted NK cells. For example, induced pluripotent stem cell (iPSC)-derived NK cells expressing high affinity non-cleavable CD16, and membrane-bound IL-15/IL-15R in combination with silenced CD38 expression have demonstrated enhanced persistence and anti-tumour functions [39].

## NK-cell therapy—an historical perspective

The observation that NK cells have cytolytic and anti-tumour activity in the mouse provided a rationale for using NK cells as immunotherapeutic agents. Studies in humans demonstrating that NK cells have intrinsic anti-tumour activities have been more challenging as genetic defects solely affecting the NK-cell lineage are relatively rare. Nevertheless, in a prospective longitudinal study of over 3000 individuals it was observed that those with low NK-cell cytotoxic activity have a higher incidence of cancer development [26]. Initial human immunotherapy studies were conducted by Rosenberg *et al*. using IL-2-activated peripheral blood mononuclear cells (PBMCs) to generate lymphokine-activated killer cells (LAKs). This cell preparation contains a heterogenous effector cell population of NK cells and T cells. Results from this study showed encouraging responses in 11 out of 25 individuals with a variety of metastatic cancers [40]. Subsequently a study by Ruggeri *et al*. more clearly demonstrated the potential for NK-cell therapy [41]. In this study, AML patients with enhanced donor-versus-recipient NK-cell alloreactivity had significantly lower levels of relapse. This concept of NK-cell alloreactivity was based on the ‘missing-self model’ of NK-cell activation, in which the donor NK cells are not inhibited by KIR engagement with the HLA-C molecules present in the recipient. This was taken forward by Miller *et al*., who used haploidentical family members to generate NK-cell products that were infused into patients with AML and maintained with low-dose subcutaneous IL-2 in the recipient [42]. Complete remissions were observed in 5 out of 19 individuals with AML, with significantly improved responses evident in patients who received KIR ligand mismatched grafts [42]. The concept of NK-cell alloreactivity has been further taken forward by the use of blocking antibodies against the MHC-I inhibitory receptors KIR and NKG2A, which is discussed further below. The alternative to blocking inhibitory receptors is to stimulate the activating receptors through antibody crosslinking and this forms the basis for the development of the NK-cell engagers (NKCE). These target antigens expressed on the cancer cell surface and NK-cell activating receptors including CD16, NKG2D, NKp30, and NKp46. This technology is evolving from engagers which have two moieties, targeting one on the NK cell and one on the tumour cell, to NKCE targeting two or more NK receptors simultaneously for enhanced potency [43]. Merging the opportunities of NK-cell adoptive transfer with tumour antigen-specific responses can be achieved using chimeric antigen receptor (CAR)-NK cell technology which is another rapidly evolving area discussed further in this article below.

## Advantages for targeting NK cells in cancer

NK cells possess multiple properties that complement CD8+ cytotoxic T-cell function against cancer. For example, while downregulation of HLA-A, -B, and -C alleles on tumour cells is a major resistance mechanism for CD8+ T cells [4], for NK cells this can be advantageous as loss of HLA-A, -B, and -C expression relieves their inhibition through the KIR. NK cells have multiple activating receptors to detect stress-induced ligands that are upregulated on tumour cells, such as MICA/B and ecto-calreticulin, and can therefore mediate killing in the absence of a specific tumour neoantigen. Furthermore, as already highlighted, NK cells promote DC recruitment and T-cell activation in tumours [21, 23, 44]. Adoptive NK-cell therapeutics also have off-the shelf capability and in clinical trials to date have not been associated with cytokine release syndrome (CRS), GVHD, or neurotoxicities which can plague T-cell therapies [45]. Inflammatory cytokines derived from activated macrophages are thought to drive CRS during T-cell therapy [46] and these cytokines remain low during adoptive NK therapy [47]. Furthermore, donor NK cells can lyse autologous donor T cells and thereby suppress T-cell-mediated GVHD [48].

**Figure 1.**
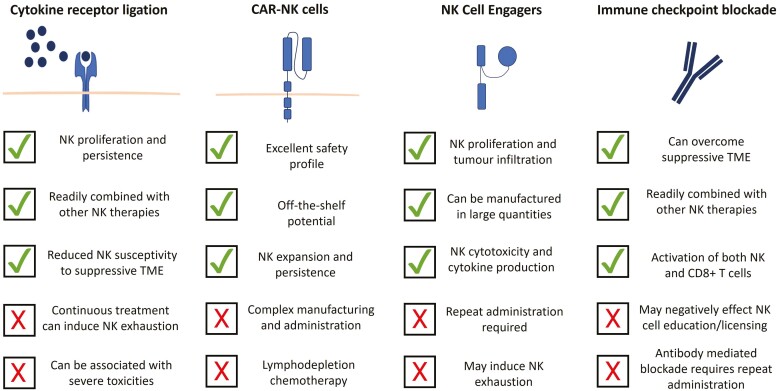
Strategies under clinical development to enhance NK-cell activation against cancer.

In this review, we highlight recent advances in the development of NK-targeted therapeutics including NK-cell engagers (NKCEs), cytokines, CAR-NK cells, and immune checkpoint inhibitors, and discuss their potential for the treatment of patients with cancer (Figure 1).

## Cytokine receptor engagement

NK-cell maturation, proliferation, and function are critically dependent upon cytokine receptor signalling. In accordance with this, the use of cytokine receptor ligation to potentiate anti-tumour NK-cell function is demonstrating promising efficacy in multiple different settings, including in combination with CAR-NK cells and NKCE strategies as described in the following sections.

The most widely targeted cytokine in development for NK-cell therapy is IL-15, with the IL-15R expressed on both NK cells and CD8+ T cells, but importantly, not regulatory T cells [49]. This is a key advantage over IL-2-based therapeutics [50] and importantly, IL-15 has been reported to prevent the loss of NK cell effector functions within the tumour microenvironment [28]. Continuous treatment of human NK cells with IL-15 can, however, induce NK-cell exhaustion [51], and therefore both the treatment schedule and route of administration must be carefully considered to achieve maximal benefit for patients. NIZ985 is a recombinant heterodimer of IL-15 and the IL-15 receptor alpha, mimicking the transpresentation of IL-15, which induces significant proliferation and IFNγ production in NK cells from patients with metastatic or unresectable solid tumours and is currently under evaluation in phase I/Ib trial in patients with advanced solid tumours or lymphoma (NCT04261439). The IL-15 superagonist complex N-803 (formerly termed ALT-803) induces potent activation of NK cells and has been shown to be safe when administered alone and in combination with the anti-CD20 antibody rituximab in patients with indolent non-Hodgkin lymphoma [52, 53]. In a phase I study, N-803 significantly increased the proliferation of NK cells, and to a lesser extent the proliferation of CD8+ T cells [52]. In accordance with this, N-803 administration in cynomolgus monkeys increased NK proliferation at lower concentrations than that required for CD8+ T-cell proliferation [54].

N-803 is currently under assessment in multiple active clinical trials, including a phase II/III trial for advanced non-small cell lung cancer in combination with the anti-PD1 antibody pembrolizumab (NCT05096663). This combination may be of particular value because the combination of N-803 with PD-1/PDL-1 blockade has recently been shown to overcome resistance to IL-15 therapy in preclinical models of ovarian cancer [55]. N-803 has also been used to support the expansion and persistence of memory-like NK cells in patients with leukaemia given reduced-intensity conditioning for HLA-haploidentical haematopoietic cell transplantation [56]. Importantly, in this study, there was over 1000-fold NK-cell expansion and NK cells persisted for over 2 months in patients [56]. This demonstrates the powerful capability of IL-15 to promote NK-cell expansion and persistence in patients. Conversely, a recent study has demonstrated that N-803 given during allogeneic cell therapy actually enhances clearance of donor NK cells by recipient CD8+ T cells [57]. This effect occurred in two independent clinical trial cohorts of relapsed/refractory AML patients treated with histocompatibility complex-haploidentical NK-cell therapy. This highlights that IL-15 receptor-mediated CD8+ cell activation must be carefully assessed to optimize IL-15 receptor-targeted therapeutics, particularly in the adoptive transfer setting. In accordance with CD8+ T-cell clearance being dependent upon MHC recognition, long-term >2-month expansion of NK cells was evident in HLA-mismatched CAR-NK-cell therapy [47]. This also highlights a possible advantage for NK persistence with autologous NK-cell therapies to circumvent CD8+ T-cell mediated clearance. Indeed, autologous NK cells have shown feasibility in treating multiple myeloma [58] and the *ex vivo* expanded autologous NK therapy (SNK01-US01) is currently under clinical evaluation for patients with solid tumours refractory to conventional therapy (NCT03941262).

It has recently been demonstrated that the *ex vivo* culture of NK cells with IL-15 in combination with nicotinamide, a vitamin B3 derivative involved in ATP generation, enhances NK-cell cytotoxicity and cytokine production, protects against oxidative stress, and, increases NK-cell persistence *in vivo* compared to IL-15 alone [59]. Furthermore, the combination of IL-15 with nicotinamide led to stable surface expression of CD62L, an L selectin critical for NK-cell adhesion and homing to the lymph nodes. In a phase 1 trial of patients with relapsed or refractory non-Hodgkin lymphoma (NCT03019666), the adoptive transfer of NK cells cultured with IL-15 and nicotinamide in combination with rituximab showed a complete response in 13 of 19 patients, with NK cells detected 14 days post-infusion [59].

The *ex vivo* stimulation of NK cells with IL-12, IL-15, and IL-18 to generate CIML NK cells has demonstrated superior anti-tumour efficacy in preclinical studies [34] and CIML NK cells are now under assessment in multiple clinical trials for both solid and haematological malignancies (Table 1). Utilization of CIML NK cells has previously involved *ex vivo* stimulation and infusion into patients. An alternative approach has recently been described however which allows NK memory cell formation *in vivo* by utilization of a fusion protein complex that combines IL-12, IL-15, and IL-18 to induce strong NK activation [60]. If transferred to the clinic, this approach would have the major benefit of utilizing CIML NK cells without the requirement for complex *ex vivo* culture conditions and adoptive transfer protocols.

**Table 1.** Selected clinical trials utilising cytokine receptor ligation to promote NK-cell activation against cancer from www.clinicaltrials.gov

Disease setting	Therapy	Clinical stage	Clinical trial number	Status	Sponsor
Advanced solid tumours and lymphoma	NIZ985 +/- spartalizumab or tislelizumab	Phase I/Ib	NCT04261439	Active, not recruiting	Novartis Pharmaceuticals
Recurrent/metastatic gastric or head and neck cancer	N-803 + PD-L1 CAR-NK Cells + pembrolizumab	Phase II	NCT04847466	Recruiting	National Cancer Institute
Advanced non-small cell lung cancer	N-803 + pembrolizumab	Phase II/III	NCT05096663	Active, not recruiting	SWOG Cancer Research Network
Advanced or metastatic non-small cell lung cancer	N-803 + current standard of care or standard of care alone	Phase III	NCT03520686	Active, not recruiting	ImmunityBio, Inc.
Relapsed acute myeloid leukaemia after HLA-matched related or unrelated allogeneic hematopoietic cell transplant	CIML NK Cell Infusion	Phase I/II	NCT03068819	Recruiting	Washington University School of Medicine
Acute myeloid leukaemia	CIML NK cell infusion + N-803	Phase II	NCT02782546	Recruiting	Washington University School of Medicine
Refractory or relapsed acute myeloid leukaemia	CIML NK cell infusion	Phase I/II	NCT05580601	Recruiting	Children’s Hospital Medical Center, Cincinnati
Advanced head and neck cancer	CIML NK cell infusion + N-803 with or without cetuximab or ipilimumab	Phase I	NCT04290546	Recruiting	Dana-Farber Cancer Institute
Relapsed or refractory multiple myeloma or relapsed/refractory CD20-positive non-Hodgkin lymphoma	Nicotinamide expanded-natural killer cell-based therapy + elotuzumab or rituximab (IL-15 during *ex vivo* expansion followed by IL-2 *in vivo*)	Phase I	NCT03019666	Completed	Masonic Cancer Center, University of Minnesota

## CAR-NK cells

CAR-T-cell therapies are now an established treatment option for patients with B-cell lymphoma, B-cell acute lymphoblastic leukaemia, or multiple myeloma [61]. However, these come at a high treatment cost and can be associated with severe toxicities. NK cells are under clinical assessment for solid and haematological malignancies in multiple phases 1 and 2 trials as an alternative cellular source for CAR-based therapies (Table 2) [45, 62]. Importantly, CAR-NK cells have the potential for off-the-shelf utility (allowing for reduced product cost and time) and reduced toxicities compared to CAR-T therapy (reviewed in [45, 63, 64]). A variety of sources of NK cells are being investigated for CAR-NK-cell production and these include induced pluripotent stem cells (iPSC), cord blood, peripheral blood from healthy donors, immortalized NK-like cell lines, and autologous cancer patient-derived NK cells [65]. Specific CAR-mediated recognition of surface tumour antigens is usually achieved by NK-cell expression of single-chain variable fragment (scFv) and the potential of CAR-NK cells to treat cancer was demonstrated in a landmark study published in 2020, in which anti-CD19 CAR-NK cells derived from cord blood were administered to 11 HLA-mismatched patients with CLL or non-Hodgkin lymphoma [47]. In this study, there were no incidences of CRS, neurotoxicity, or GVHD and furthermore, an overall 73% response rate was achieved, with complete remissions in 7/11 patients at a median follow-up of 13.8 months. Importantly, expansion of CAR-NK cells was evident following infusion and CAR-NK cells could be detected for at least 12 months by PCR [47] demonstrating the potential for long-term persistence of CAR-NK cells. Interestingly, in the two patients available for assessment of lymph nodes, CAR-NK cells preferentially homed to the lymph nodes compared to the blood and bone marrow [47] indicating that CAR-NK cells may be home to sites with high tumour burden.

**Table 2.** Selected clinical trials for CAR-NK cells in solid and haematological malignancies from www.clinicaltrials.gov

Disease setting	Target antigen	Cellular source	Clinical phase	Clinical trial number	Status	Sponsor
Advanced solid tumours	Claudin6	Autologous PBMC	Phase I/II	NCT05410717	Recruiting	Second Affiliated Hospital of Guangzhou Medical University
Relapsed or refractory haematological malignancies	CD70	Cord blood	Phase I/II	NCT05092451	Recruiting	M.D. Anderson Cancer Center
Relapsed or refractory B-cell non-Hodgkin lymphoma	CD19	Cord blood	Phase II	NCT05020015	Recruiting	Takeda
Recurrent/metastatic gastric or head and neck cancer	PD-L1	Engineered NK-92 cells	Phase II	NCT04847466	Recruiting	National Cancer Institute
Relapsed or refractory B-cell lymphoma or chronic lymphocytic leukemia	CD19	iPSC	Phase I	NCT04245722	Active, not recruiting	Fate Therapeutics
Relapsed or refractory multiple myeloma	BCMA	iPSC	Phase I	NCT05182073	Recruiting	Fate Therapeutics
Acute myeloid leukaemia	CD33/ CLL1	Information not available	Phase I	NCT05215015	Recruiting	Wuxi People’s Hospital
Relapsed and refractory multiple myeloma	BCMA	NK92	Phase I/II	NCT03940833	Unknown	Asclepius Technology Company Group (Suzhou) Co., Ltd.
Recurrent HER2-positive glioblastoma	HER2	NK-92/5.28.z	Phase I	NCT03383978	Recruiting	Johann Wolfgang Goethe University Hospital
Relapsed or refractory CD19-positive B-cell malignancies	CD19	iPSC	Phase I	NCT05336409	Recruiting	Century Therapeutics, Inc.
Recurrent or refractory CD19 positive B-cell malignant tumours	CD19	Information not available	Phase I	NCT05410041	Recruiting	Beijing Boren Hospital
MUC1 positive advanced refractory or relapsed solid tumours	MUC1	Information not available	Phase I/II	NCT02839954	Unknown	PersonGen BioTherapeutics (Suzhou) Co., Ltd.
Locally advanced or metastatic pancreatic cancer	PD-L1	Engineered NK-92 cells	Phase II	NCT04390399	Recruiting	ImmunityBio, Inc.
Patients who have previously received treatment with PD-1/PD-L1 immune checkpoint inhibitors	PD-L1	Engineered NK-92 cells	Phase II	NCT03228667	Active, not recruiting	ImmunityBio, Inc.

An important mechanism for relapse during CAR-T-cell therapy is the loss of CAR-target antigen expression. CAR-NK cells can potentially kill tumour cells independently of the CAR-target antigen via their retention of native activating receptors and via ADCC in combination with antibodies that target different tumour associated antigens. For example, anti-CD19 CAR-NK cells obtained from iPSC engineered to express high affinity non-cleavable CD16 have demonstrated potent anti-lymphoma efficacy in murine models and were able to target both CD19- and CD19+ target cells in combination with anti-CD20 antibodies [66]. In addition, a multi-antigen targeting strategy has been assessed for multiple myeloma, with anti-BCMA CAR-NK cells engineered with a high-affinity non-cleavable CD16 demonstrating strong efficacy in murine models of myeloma in combination with the anti-CD38 antibody daratumumab [67]. This study also demonstrated the importance of minimizing NK-cell fratricide to enhance CAR-NK-cell efficacy, with the silencing of CD38 expression able to inhibit CAR-NK-cell fratricide during combination with daratumumab [67]. An alternative strategy developed to reduce CAR-NK-cell fratricide is via the co-expression of an inhibitory CAR construct which recognizes an antigen expressed on NK cells [68]. This dual CAR approach allowed for CAR-NK mediated lysis of tumour cells that expressed the tumour antigen, while CAR-NK cells which co-expressed the target antigen (via trogocytosis) and the target antigen for the inhibitory CAR were spared from lysis. Importantly, this approach enhanced CAR-NK-cell persistence and efficacy in pre-clinical models [68] demonstrating its strong potential for translation to the clinic.

In patients with solid tumours, intracranial injection of anti-HER2 CAR-NK cells in patients with glioblastoma demonstrated that CAR-NK cells are safe and well tolerated when given by this route and can have clinical activity, with stable disease being the best response in this phase 1 trial [69]. Anti-GD2 CAR-NK cells have also been shown to inhibit tumour growth in a xenograft murine model of paediatric brainstem gliomas [70], highlighting the potential utility of CAR-NK cells for brain tumours which are notoriously hard to treat effectively. In addition to these tumour types, CAR-NK cells are now under clinical evaluation in early-phase trials for patients with a number of different solid tumours, including head and neck cancer and pancreatic cancer (Table 2).

NK cells have also been engineered to express a functional T-cell receptor (TCR)/CD3 construct, as opposed to a scFv, termed TCR-NK cells [71]. This allows for NK-cell-mediated targeting of specific peptide: MHC cancer-associated antigens and targeting of antigens that are unsuitable for scFv CAR NK cells. Examples of TCR-NK-cell therapeutics under investigation include targeting the MAGE-A4 antigen for solid tumours [72] and the NY-ESO-1 antigen in multiple myeloma [73]. Building on this approach, dual expression of a TCR specific to human papillomavirus E7 antigen in combination with a CAR targeting the trophoblast cell surface antigen 2 (TROP2) has been shown to synergistically enhance NK-cell function compared to TCR-NK cells alone in preclinical models [74].

Using single-cell RNA sequencing and mass cytometry in combination with murine models and patient samples, Li *et al*. recently demonstrated that loss of metabolic fitness of CAR-NK cells is a major cause of tumour resistance to therapy [75]. Incorporation of IL-15 into the CAR construct improved NK-cell function. However, over time these CAR+IL-15-NK cells still lost their anti-tumour functions against highly metabolic tumours, suggested to be due to local depletion of nutrients by the tumours [75]. Importantly, a second infusion of CAR+IL-15-NK cells showed increased efficacy and allowed for more effective control of tumours [75] demonstrating that the treatment schedule of CAR-NK cells will need to be carefully assessed to optimize patient outcomes. This work is in agreement with previous studies of patients treated with CAR-NK cells, which demonstrated that the metabolic fitness of CAR-NK cells is an important determinant of clinical outcome [75].

An alternative approach to enhance CAR-NK-cell function includes the targeting of the cytokine-inducible SH2-containing (CISH) protein, a crucial inhibitory regulator of IL-15-mediated signalling. This strategy led to augmented metabolic fitness of CAR-NK cells, increased expansion, more effective lysis of tumour cells *in vitro,* and enhanced anti-tumour efficacy *in vivo* [76, 77]. The utilization of protocols that generate CIML has been applied to CAR technology, making CAR-memory-like NK cells. This enhances CAR-NK persistence and multiple studies have demonstrated the promising efficacy of this approach, including for AML [78] and lymphoma [79] and solid tumours including head and neck cancer [38]. Furthermore, CMV-induced memory-like NK cells typically lack SYK expression and it has recently been shown that CRISPR-Cas9 silencing of SYK enhances NK-cell cytotoxicity and cytokine production, indicating that genetic engineering approaches to recapitulate key features of memory-like NK cells could potentially be used to improve CAR-NK-cell function [80]. Furthermore, CAR-NK-cell function may be improved by rational drug combination strategies such as epigenetic modulators, oncolytic viruses, small molecule inhibitors, [63] or with stimulator of interferon genes (STING) agonists which have recently been demonstrated to potentiate CAR-NK-cell killing of tumour cells [81, 82]. Additional promising approaches to improve CAR-NK-cell function include: tailoring the intracellular signalling proteins within the CAR construct for NK cells rather than using a CAR construct designed initially for T cells [83]; and optimizing the affinity of the CAR for the target antigen to minimize on-target off-tumour cytotoxicity [84, 85].

## NK-cell engagers (NKCEs)

NK cells express multiple activating receptors which can synergize to enhance the level of NK cell activation. The FcγRIII receptor (CD16) allows NK cells to mediate ADCC and thus contribute to the therapeutic efficacy of tumour targeting antibodies [18, 86–89]. To exploit this, a new generation of antibody constructs, termed NK-cell engagers (NKCEs) are now under development which combine antibody variable domains that bind tumour antigens with antibody domains that bind NK cell receptors. Receptors targeted alone or in combination with this strategy include CD16, NKp30, NKp46, NKG2D, or cytokine receptors [90]. Therefore NKCEs which simultaneously engage one or more activating receptors and/or cytokine receptors on NK cells allow for more potent activation against tumour cells. This can enhance NK-cell proliferation compared to ligation of CD16 alone [90, 91]. These bi-specific (BiKE), tri-specific (TriKe), and tetra-specific NKCEs are under development against both haematological malignancies and solid tumours, with examples of clinical trials for these shown in Table 3.

**Table 3.** Selected clinical trials for NKCEs in cancer from www.clinicaltrials.gov

Disease	Tumour antigen target	NKCE	Clinical phase	Clinical trial number	Status	Sponsor
Relapsed or refractory acute myeloid leukaemia, B-cell acute lymphoblastic leukemia, or high risk-myelodysplasia	CD123	SAR443579	Phase I/II	NCT05086315	Recruiting	Sanofi
Relapsed or refractory multiple myeloma, relapsed or refractory light-chain amyloidosis	BCMA	SAR445514	Phase I/II	NCT05839626	Recruiting	Sanofi
Relapsed or refractory acute myeloid leukaemia	CD123	AFM28	Phase I	NCT05817058	Recruiting	Affimed GmbH
Advanced solid malignancies	EGFR	AFM24	Phase I/II	NCT04259450	Active, not recruiting	Affimed GmbH
Relapsed or refractory CD30-positive T-cell lymphoma	CD30	AFM13	Phase II	NCT04101331	Active, not recruiting	Affimed GmbH
Advanced or metastatic EGFR-expressing cancers	EGFR	AFM24	Phase I/II	NCT05099549	Active, not recruiting	NKGen Biotech, Inc.
Recurrent or refractory CD30-positive Hodgkin or non-Hodgkin lymphomas	CD30	AFM13	Phase I/II	NCT04074746	Active, not recruiting	M.D. Anderson Cancer Center
CD33-expressing high-risk myelodysplastic syndromes, refractory/relapsed acute myeloid leukaemia, or advanced systemic mastocytosis	CD33	GTB-3550	Phase I/II	NCT03214666	Terminated due to development of the second-generation TriKE GTB-3650	GT Biopharma, Inc.
Relapsed and/or refractory multiple myeloma	BCMA	CC-92328	Phase I	NCT04975399	Recruiting	Celgene
Advanced solid tumours	HER2	DF1001	Phase I/II	NCT04143711	Recruiting	Dragonfly Therapeutics
Relapsed or refractory acute myeloid leukaemia	CD33	CC-96191	Phase I	NCT04789655	Recruiting	Celgene

NKCEs have been tested predominantly against haematological malignancies, targeting antigens such as CLEC12A, CD19, CD20, CD33, CD123, and BCMA. The benefit of targeting multiple NK receptors simultaneously is exemplified by an NKCE targeting CD16, NKp46, the β-chain of the interleukin-2 receptor (IL-2R), and CD20 [91]. This tetra-specific NKCE showed significantly improved control of lymphoma growth in a murine model compared to the anti-CD20 therapeutic antibody obinutuzumab. Importantly, the incorporation of the IL-2R stimulating domain induced NK-cell proliferation *in vivo* and led to increased NK infiltration into lymphoma tumours [91]. In this NKCE, NKp46/CD16 and tumour antigen engagement induced strong NK cytotoxicity, while IL-2R ligation was required to drive NK expansion and infiltration [91]. This highlights the benefit of incorporating cytokine receptor ligation into NKCE development. Incorporation of an IL-15 moiety into a TRiKE targeting CD16 and CD19 has also been shown to induce NK-cell expansion and to improve the killing of CLL cells compared to rituximab [92]. In AML, a TriKe targeting the activating receptor NKG2C, the IL-15R, and the tumour-associated antigen CD33 showed potent NK-cell degranulation and cytokine production in response to primary CD33+ AML blasts [93]. Targeting the myeloid lineage antigen CLEC12A in combination with CD16 and IL-15R ligation by a TriKe stimulated NK-cell proliferation and cytotoxicity against primary AML blasts whilst sparing normal haematopoietic stem cells [94]. Furthermore, in a patient-derived xenograft model, this TriKe reduced AML burden in the bone marrow of mice [94].

A TriKe targeting NKp46, CD16, and CD20 provided superior efficacy against CD20+ lymphoma cells *in vivo* and *in vitro* compared to obinutuzumab, an approved anti-CD20 therapeutic antibody [95]. Against pediatric B-ALL cells which express low levels of CD20, a CD19 targeting NKCE which ligates CD16 in combination with either NKp46 or NKp30 showed potent activation of NK cells against primary B-ALL cells and could overcome inhibitory HLA-mediated interactions [96]. Furthermore, targeting NKp46, CD16, and the tumour-associated antigen CD123 (IPH6101/SAR’579) induced NK-cytokine secretion and activation against primary AML cells [97] demonstrating improved efficacy *in vivo* compared to an ADCC-enhanced therapeutic antibody targeting CD123 [97]. IPH6101/SAR’579 is now in a phase 1/2 trial for patients with relapsed or refractory AML, B-ALL, or high risk-myelodysplastic syndrome (NCT05086315), with initial reports indicating good tolerance and clinical benefit in patients with relapsed/refractory AML [98]. Furthermore, the NKCE IPH6401/SAR’514 targeting BCMA, CD16, and NKp46 is in a phase 1/2 trial for patients with relapsed/refractory multiple myeloma or light-chain amyloidosis (NCT05839626). Other NKCEs under assessment in haematological malignancies included a TriKE (GTB-3550) targeting CD16, IL-15, and the tumour-associated antigen CD33 in AML and high-risk myelodysplastic syndromes in a phase 1 trial (NCT03214666). This therapeutic is now being superseded by the second-generation camelid nanobody NKCE GTB-3650.

Administration of NKCEs into cancer patients relies on the effector mechanisms of patient NK cells which can be dysregulated and or have low frequency. A promising strategy to overcome this is to combine NKCEs with adoptive transfer of healthy donor-derived *ex vivo* expanded NK cells. For example, combining the tetravalent bispecific engager AFM13 which targets CD30 on lymphoma cells and CD16 on NK cells, with pre-activated cord blood-derived NK cells demonstrated improved control of CD30+ lymphoma *in vivo* compared to AFM13 or pre-activated NK cells alone [99]. AFM13 in combination with cord blood-derived NK cells is now under assessment in a phase I/II trial for patients with recurrent or refractory CD30+ Hodgkin or non-Hodgkin lymphoma (NCT04074746). Donor selection may also be important and ‘superdonors’ with large subpopulations of NKG2C+ adaptive NK cells provide a ready source of allogeneic NK cells which express single inhibitory KIR, such that they would be predicted to be potently alloreactive. These have been combined with an anti-CD33 TriKE to good effect in a preclinical AML model [16].

NKCEs have also been studied in solid tumours, but less extensively. For instance, a CD16/IL-15R/HER2 targeting TRiKE induced NK-cell proliferation and demonstrated promising efficacy against ovarian cancer cells *in vitro* and *in vivo* [100]. A CD16/IL15R/mesothelin TriKE enhanced the proliferation, cytotoxicity, and cytokine production of patient-derived NK cells against lung cancer cell lines [101]. In addition, this NKCE significantly improved control of tumour growth in a metastatic xenograft model [101]. Furthermore, to overcome NK-cell dysfunction in patients, the combination of NKCE with adoptively transferred NK cells in solid tumours is currently under clinical assessment (Table 3). For example, the autologous NK-cell product SNK01 in combination with AFM24, a CD16 and EGFR engager, is currently in a phase I/II trial for patients with advanced or metastatic ERGF-expressing solid tumours (NCT05099549).

In addition to targeting antigens specifically associated with tumour cells, NKCEs have also shown utility against antigens that are shared by both tumour cells and tumour-associated stromal cells [102]. This is important because tumour-associated stromal cells can aid tumour growth and mediate immunosuppression [102], and therefore targeting these cells may be advantageous by modulating the tumour microenvironment, especially for solid tumours which can rapidly cause NK dysfunction [28]. An example of this is TEM8, which is expressed by tumour associated stromal cells, fibroblasts, endothelial cells, and various cancer types including breast cancer cells. A CD16/IL-15R/TEM8 targeting TRiKE stimulated NK-cell cytotoxicity against both tumour cells and tumour endothelial cells *in vivo,* with a decrease in endothelial density in tumours evident [102]. Interestingly, this NKCE was also able to increase NK-cell infiltration into subcutaneous tumours formed from breast cancer cell lines compared to functionally equivalent IL-15 and could enhance the proliferation of NK cells [102]. This study reveals the possibility of using NKCE to target not only the tumour cells themselves but also the surrounding tumour microenvironment and angiogenesis to enhance their efficacy.

## Immune checkpoint blockade

NK-cell activation is tightly regulated by an array of inhibitory receptors and disruption of these receptor: ligand interactions can release NK cells from immunosuppression in cancer. The KIR family of receptors is a critical component of NK recognition of self and plays an important role in NK-cell education [2]. The inhibitory KIR recognize HLA-A/B/C and disruption of KIR: HLA interactions allows for potent NK-cell activation against target cells. However, the KIR have proved difficult to target effectively to date. Innate Pharma developed the anti-KIR blocking antibody lirilumab to potentiate NK-cell effector functions and this approach showed promising preclinical activity *in vitro* and *in vivo* with enhanced NK-cell cytotoxicity and ADCC [103]. However, in phase 2 clinical trial for patients with multiple myeloma lirilumab was ineffective, reducing NK-cell function and KIR2D expression by trogocytosis [104, 105]. Further evaluation of lirilumab is currently ongoing in a phase 2 clinical trial for squamous cell carcinoma of the head and neck in combination with the anti-PD-1 antibody nivolumab (NCT03341936) and these results are eagerly anticipated.

NK cells are under constitutive inhibition by inhibitory receptors for MHC class I, and genome-scale CRISPR-based gene editing screens have revealed that HLA-E interactions with NKG2A are a key determinant for tumour cell sensitivity to NK-cell mediated lysis [106]. Upon ligation, NKG2A suppresses NK-cell activation via SHP-1/2 making it an attractive target to release NK cells from inhibition [107]. HLA-E is widely expressed in solid tumours and haematological malignancies [108, 109] and can be further increased upon IFNγ stimulation [110, 111], or by lymph node associated signals on CLL cells [112]. Furthermore, NKG2A-positive cells are detected within solid tumour tissues [108], and in the lymph nodes of patients with lymphoma [113]. Importantly, it has recently been shown that circulating tumour cells are protected from NK-cell mediated clearance via NKG2A:HLA-E interactions, with HLA-E expression promoted by platelet-derived RGS18 and NKG2A blockade able to restore NK function and prevent tumour metastasis *in vivo* [114]. In accordance with this, NKG2A+ NK cells are detected in tumour draining lymph nodes, the first site for metastases in patients with breast cancer [115]. Additionally, disruption of NKG2A function may have particular importance in combination with adoptive NK-cell therapies because clinically relevant NK-cell expansion techniques lead to significantly increased surface NKG2A expression (reviewed in [116]).

The NKG2A blocking antibody monalizumab promotes NK-cell effector function *in vitro* and *in vivo* against tumour cells and enhances ADCC, as well as the efficacy of cancer vaccines [108, 117]. Monalizumab is currently in several clinical trials for solid tumours, including a phase 3 clinical trial in combination with durvalumab (anti-PD-L1) in patients with unresectable, stage III non-small cell lung cancer (NCT05221840) (Table 4). However, a trial of monalizumab and cetuximab in head and neck cancer was recently discontinued. NKG2A is also expressed on CD8+ T cells [108, 117] and NKG2A expression identifies a subset of the innate-like Vδ2 T cells with enhanced anti-tumour functions [118]. This indicates that NKG2A blockade in patients may simultaneously release the effector functions of NK cells as well as CD8+ T cells and Vδ2 T cells. In addition to monalizumab, the NKG2A blocking antibody BMS-986315 is currently in a phase 1/2 trial for patients with advanced solid tumours either alone or in combination with nivolumab or cetuximab (NCT04349267). In addition to direct antibody-mediated blockade of NKG2A, other approaches under investigation include antibody-mediated blockade of HLA-E, as well as CRISPR-Cas9 mediated silencing of NKG2A expression [116, 119, 120]. Interestingly, tumour cell surface expression of HLA-E is sensitive to downregulation by various clinically relevant pharmacological agents such as the XPO1 inhibitor selinexor, the proteosome inhibitor bortezomib, and the cyclin-dependent kinase inhibitor dinaciclib [112, 121–123]. This indicates that the combination of NK cells with approved small molecules which modulate HLA-E levels on tumour cells may have synergistic anti-tumour effects.

**Table 4.** Selected clinical trials for immune checkpoint inhibitors in cancer from www.clinicaltrials.gov

Disease	Inhibitory receptor target	Antibody	Clinical phase	Clinical trial number	Status	Sponsor
Locally advanced, unresectable non-small cell lung cancer, who have not progressed following platinum-based cCRT	NKG2A	Monalizumab	Phase III	NCT05221840	Recruiting	AstraZeneca
Resectable, early-stage non-small cell lung cancer	NKG2A	Monalizumab	Phase II	NCT05061550	Recruiting	AstraZeneca
Advanced solid tumours	NKG2A	BMS-986315	Phase I/II	NCT04349267	Recruiting	Bristol-Myers Squibb
Recurrent squamous cell carcinoma of the head and neck	KIR2DL1/2/3	Lirilumab	Phase II	NCT03341936	Active, not recruiting	Dana-Farber Cancer Institute

CISH is a critical negative regulator of IL-15 signalling in NK cells which acts via inhibition of JAK1 enzymatic activity [124, 125]. CRISPR-Cas9 mediated silencing of CISH in human NK cells enhances cytokine production capacity and NK cytotoxicity against K562 cells and improves the control of tumours in murine models [126]. Importantly, however, CISH-deleted NK cells are still sensitive to TGFβ mediated suppression and the combined suppression of both CISH and TGFβ substantially improved anti-tumour immunity compared to suppression of either protein alone [127]. In glioblastoma multiforme (GBM), the release of TGFβ from glioblastoma stem cells was mediated by αv integrins and the targeting of αv integrin or blockade of the TGF-β receptor improved NK-cell function and improved tumour control in a murine model [128]. TGFβR ligation induces SMAD2/3-mediated NK-cell suppression and interestingly, TGFβ-independent suppression of NK cells via SMAD2/3 has also been identified. SMAD2/3-mediated suppression of NK cells occurred via activin-A mediated ligation of the type I activin receptor ALK4 that is expressed on NK cells [129]. This highlights the requirement to consider the contribution of other receptors in addition to TGFβR to fully overcome SMAD2/3-mediated suppression of NK function. Furthermore, hypoxia is a characteristic feature of the tumour microenvironment and NK cells are more sensitive to hydrogen peroxide compared to T or B cells due to decreased expression of peroxiredoxin-1 (PRDX1) [130]. In accordance with this, overexpression of PRDX1 can augment NK cell and CAR-NK-cell anti-tumour activity under oxidative stress conditions [130].

The immune checkpoint receptors PD-1 and TIGIT are classically targeted to augment T-cell function and both TIGIT and PD-1 are expressed in NK cells. TIGIT is associated with NK-cell exhaustion [131] and TIGIT blockade augmented NK-cell activation and CD8+ T-cell effector function in murine models of cancer [131]. Furthermore, expression of functional PD-1 is detected on both resting and activated human NK cells [132, 133], and a single chain fragment of pembrolizumab increased NK cell cytokine production up to 4-fold [132]. In accordance with this, murine models have shown that NK cells are required to mediate the full therapeutic efficacy of PDL-1 blockade [133] and iPSC-derived NK cells can co-operate with T cells in combination with anti-PD1 antibodies to control tumour growth *in vivo* [20]. In cancer patients, NK-cell frequency associated with stimulatory dendritic cells, the response to anti-PD-1 therapies, and overall survival [23]. In addition, it has recently been identified that NK-cell production of the DC chemoattractant CCL5 is important for the response to anti-PD-1 therapy [44]. This highlights the important role of NK cells not only in the direct lysis of tumour cells but also in their ability to shape the adaptive anti-tumour immune response via cross-talk with DC and CD8+ T cells [21].

## Conclusions and future perspectives

NK cells hold significant potential for cancer immunotherapy due to their excellent safety record, potent cytotoxic activities, and ability to kill tumour cells with downregulated MHC expression. The unique features of NK cells also make them attractive for either combination with T-cell-directed therapies, or for use in T-cell therapy refractory settings. The potential for adoptive NK-cell therapies to provide an off-the-shelf product also provides the opportunity for reduced cost and production time compared to CAR-T cells. Specific challenges exist for different cancer types, and these will need to be assessed in pre-clinical and clinical studies to optimize anti-cancer NK-cell immunity in patients.

Therapeutic strategies which enhance the expansion, persistence, and infiltration of NK cells into areas of high tumour burden will be crucial to realize the full potential of NK cells. This is of particular importance in patients with solid tumours, with pre-clinical and clinical evidence indicating that NK cells have impaired tumour infiltration, as well as reduced activation within the immunosuppressive tumour microenvironment. In addition, sensitization of tumour cells to NK-cell killing in a drug combination strategy, as has recently been shown with the BH3-mimetic venetoclax [134], may prove useful to augment the efficacy of NK therapies. Furthermore, mRNA-encoded cytokines can enhance NK-cell activation and promote the control of tumours *in vivo* [135], highlighting the promising potential for mRNA-based therapeutics to improve NK-cell activity in cancer patients. Finally, because the chronic stimulation of NK cells without concomitant negative receptor signalling can induce exhaustion of NK cells [136], therapeutic strategies must be based on a deep understanding of NK-cell biology to realize their full potential against cancer.

## Data Availability

No new data were generated or analysed in support of this research.
